# The root rhizosphere as a functional analog to the gut microbiome: Cases for microbial symbiosis and dysbiosis in parallel contexts

**DOI:** 10.1093/pnasnexus/pgag132

**Published:** 2026-04-25

**Authors:** C Ryan Penton, Gupta Vadakattu

**Affiliations:** College of Integrative Sciences and Arts, School of Applied Sciences and Arts, Arizona State University, Mesa, AZ 85212, USA; CSIRO Agriculture and Food, Locked Bag 2, Glen Osmond, SA 5064, Australia

**Keywords:** dysbiosis, rhizosphere, human gut, microbiome, plant

## Abstract

Microbiomes associated with both the human gut and plant root rhizosphere are essential for the maintenance of host health and function as holobionts where both the host and microbiome operate as an integrated unit. Though substantial differences exist in both host biology and environment, these systems share functional parallels: both are enriched by host-derived nutrients, undergo successional shifts during development, and maintain core microbiomes that are taxonomically variable yet functionally redundant. Central to both systems is the balance that is maintained where beneficial microbes regulate nutrient cycling, modulate host immune response, and suppress pathogens in the presence of biotic and abiotic influences that may serve to disrupt this equilibrium. When dysbiosis occurs, there is a disruption in the composition and/or function of the associated microbiome and a loss of beneficial functional guilds, which results in a reduction in host fitness. These shared dynamics underscore dysbiosis as a cross-kingdom pathology that may be treated with similar interventions. Probiotics and prebiotics mirror microbial inoculants and organic amendments; synbiotics incorporate both biotic and abiotic factors, while fecal and soil microbiome transplants represent parallel strategies to restore a beneficial microbiome. By framing dysbiosis within a “One Health” perspective and illustrating the connectedness between human and plant health, this review advocates for microbial stewardship as a unifying strategy to mitigate disease, enhance resilience, and ensure sustainable health across both systems.

## Overview

A thin layer of soil, strongly influenced by a combination of plant root exudates and biotic interactions, referred to as the rhizosphere, harbors a remarkable microbiome that serves as an interface between plants and their surrounding environment. Here, within the plant rhizosphere, dynamically complex communities of bacteria, archaea, fungi, protozoa, oomycetes, viruses, and other soil fauna directly impact plant physiology and health, modulate immune responses, regulate nutrient uptake, and confer resilience/resistance against environmental stressors (1–3). Residing within this complex microbiome are beneficial bacteria and fungi that can shape the composition and diversity of the microbiome as well as mediate interactions between microbes and root surfaces and, directly or indirectly through resource competition, antagonize soilborne pathogens. These microbial interactions are not only limited to the plant rhizosphere but also include those that migrate into plants (endophytes) and those that reside on leaf surfaces (phyllosphere) (4, 5).

Although surficially different, the human gut microbiome exhibits remarkable similarities to the plant root rhizosphere with the community influenced by and influencing the health of its host through a long history of co-evolution (6). In both systems, the local environment constrains both compositional and functional diversity which results in tightly networked microbiomes that are spatially and temporally organized (7). In this context, both can be referred to as “holobionts” where the host and microbiota form a singular, functional unit (8–10). However, this connectivity can be interrupted when changes to the host or the microbiome take place. When this occurs, both the gut and rhizosphere microbiomes are susceptible to dysbiosis, a term first properly utilized by Arthur Scheunert (11), though likely first described, but not explicitly defined, by Élie Metchnikoff (12). Definitions and/or uses of the term “dysbiosis” vary significantly across writings, an issue addressed in depth by the review of Hooks and O’Malley (13). Dysbiosis has frequently been defined as changes in microbial taxonomic or functional composition, often without cause or effect evidence toward the host’s physiological response/fitness. In this review, we utilize this term as a communication tool. Whereas microbial resilience is generally considered as a key characteristic of healthy microbiomes in both the plant rhizosphere and gut microbiome systems, the term “dysbiosis” is used here when patterns or imbalances of microbiomes (structure and/or function) are linked (usually without cause-effect) to diseased or unhealthy states (13). Despite recognition of the similarities between the human gut and plant rhizosphere systems, dysbiosis in terms of key drivers, both intrinsic and extrinsic factors, and biomarkers has mostly been discussed independently (14). Since “dysbiosis” is a multifactor influenced trait where participants and the environment interact both internally and externally, a comprehensive understanding of these interactions can allow for the identification of potential strategies for interventions at different focal points, a focus of this review.

Previous reviews have discussed comparisons between the gut microbiome and plant rhizosphere microbial communities, with differing emphasis. Most recently, Ilyaskina et al. (15) modeled the human gut as an “inside out” version of the rhizosphere while highlighting functional and ecological similarities with a particular focus on shared metabolic pathways, metabolites, and receptors. Possible connectivity, in terms of the transmission of microbes from “soil to plants to the human gut” with specific examples, was the focus of a review by Ma et al. (16), while Paasch and He (17) focused on *Arabidopsis* and microbial homeostasis. In a short review, Mendes and Raaijmakers (18) discussed comparisons between microbial composition and linkages to “re-biosis,” defined there as a “re-establishment of a healthy, complex microbiome following dysbiosis.” Ramírez-Puebla et al. (19) provided an overview of how root and gut bacteria regulate host gene expression, nutrient uptake, pathogen defense, and bacteria–host interactions. This manuscript builds on previous reviews by emphasizing both microbiome and host-related factors, including microbial and system resilience, and then delves into how dysbiosis affects host health in both host–microbiome systems. Particular emphasis is placed on analogous approaches to treating dysbiosis in both human and plant systems. In the context of “One Health,” our review focuses on the human gut microbiome with comparisons that span a wide range of plants.

## A comparison of gut and rhizosphere habitats and microbiomes

Despite the evolutionary gulf between plants and humans, their microbiomes (rhizosphere and gut) overlap in their “ecological” functions. Much of what we know about the human gut microbiome is based on studies of the fecal community. While there is no direct equivalent in plants, the community associated with the rhizosphere is the closest analog. They both harbor similar functional guilds that respond to and impact the health of their host (19) and contain the highest microbial densities of any habitat from either organism (Table 1). These dense communities are made possible by host-derived energy sources (e.g. carbon) and nutrient enrichment that satisfies most macro- and micronutrient needs of microorganisms. Both microbiomes are shaped over time, with the gut microbiome evolving from birth to death, while the plant microbiome shifts from the seedling stage to senescence or death. In the gut, vertical transmission begins at birth, influenced by the birthing method (C-section versus vaginal birth) and breastfeeding (versus formula) which establishes the early infant microbiome (46), with some argument (47). In plants vertical transmission occurs via seed endophytes (48, 49), with external factors influencing subsequent colonization. For both ecosystems, the resident microbiome is also influenced by both biotic and abiotic factors as well as competition between individuals.

**Table 1:** Key similarities between the human gut and plant rhizosphere microbiomes.

Feature	Human gut	Plant rhizosphere	Interpretation
High microbial loading	>10^11^ cells/g in colon (20, 21)	>10^9^ cells/g soil (22)	Dense microbial communities due to host-derived resources
High-energy source and nutrient environment	Mucus, dead epithelial cells, dietary residues, metabolic by-products (23, 24)	Root exudates, hormones, signal molecules, mucilage, sloughed root cells (25, 26)	Both are “hotspots” of microbial activity due to nutrient enrichment
Host selected microbiome	Host genotype, mucosal immunity (27, 28)	Plant genotype, root exudates (C type, signaling molecules) (29–31)	Host plays principal role in shaping the microbiome
Functional guilds	Mucin degraders, fermenters, producers of beneficial SCFAs (32, 33)	Root decomposers, N-fixers, plant growth promoters (phytohormone production) (4, 34, 35)	Functional redundancy, similar ecological roles (SCFAs vs. phytohormones)
Community succession	Age dependent (infant to adult), food type (36)	Developmental stage (seedling to senescence) (37)	Both undergo succession based on development and environment
Impact on host health	Reduces pathogen colonization success, modulates immune system, disease resistance (38–40)	Increases stress tolerance, reduces soilborne pathogen success, disease resistance, immune modulation (41, 42)	Both exhibit bi-directional interactions that serve to shape host physiology/health
Spatial structure	Lumen vs. mucosal communities (43)	Selection from bulk soil to rhizosphere–rhizoplane–endosphere (44, 45)	While similar, spatial compartmentalization is more defined in plants

### Physiochemical gradients

**Figure 1:**
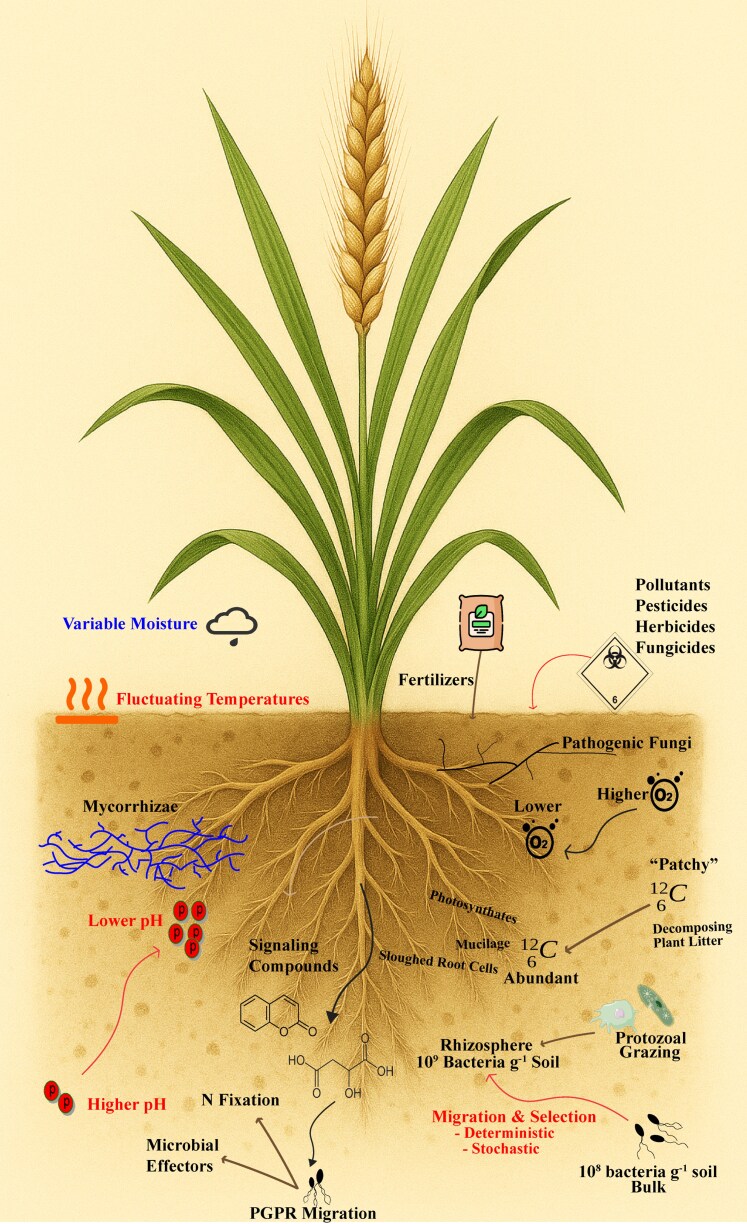
Conceptual illustration of the root rhizosphere and bulk soil illustrating factors that influence the composition and function of the microbiome.

Both microbiomes are influenced by the presence of gradients, principally related to distance from the gut epithelial cells and the root surface. The rhizosphere is shaped by O_2_, water, and pH gradients that vary with increasing distance from the root rhizoplane as well as along the transverse axis of the root (25) (Fig. 1). In this environment, O_2_ availability varies widely, with anaerobic conditions possible near the root surface and within soil aggregates due to soil texture (clay content) and the combined respiratory activity of roots and microbes (50). Aerobic conditions tend to improve with increasing distance from both the root surface and the center of aggregates such that a substantial proportion of the root zone is typically aerobic. However, aerobic status is highly dynamic, being influenced by night/day root respiration, root exudates, nutrient pulses, and soil moisture. Gradients of pH also exist with increasing acidity toward the root surface due to the exudation of protons and organic acids (51, 52). Soil temperature is also highly variable within this region, which influences moisture availability. Overall, the composition and diversity of rhizosphere communities are shaped not only by the proximity to the root surface but also by soil texture, plant type, and root architecture, among others. Microbial communities in the rhizosphere are connected with those in the surrounding bulk soil, which harbors a vast reservoir of microbial diversity, across these gradients. Selection and exclusion processes occur along the gradients toward the root surface, which contributes to distinct microbial assemblages.

**Figure 2:**
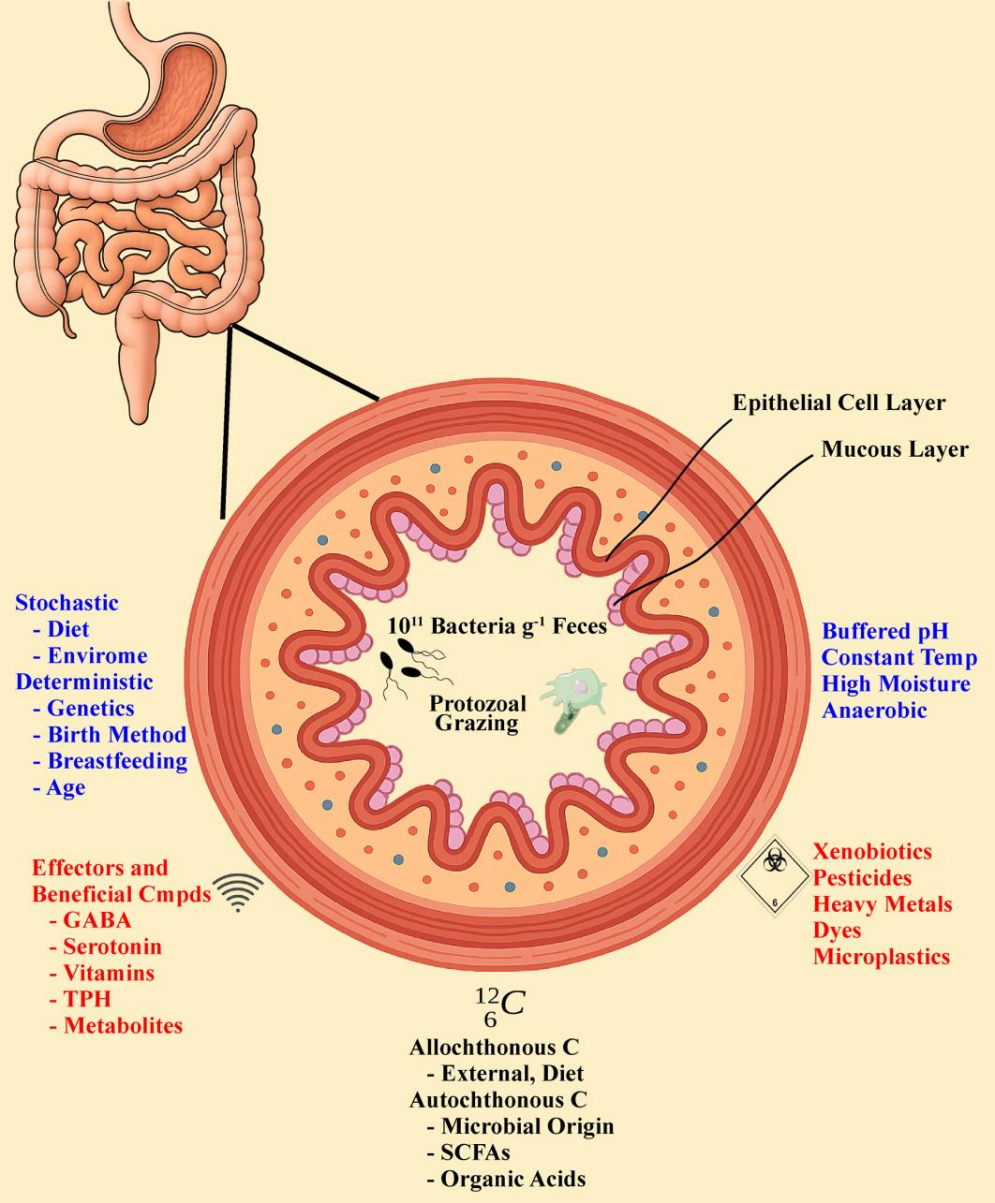
Illustration of the internal and external factors that influence the stability of the gut microbiome.

In the human gut, microbial communities are most influenced by their location either within the gut lumen or proximal to the gut epithelial cells in the associated mucosa (43) (Fig. 2). Although the upper gastrointestinal tract tends to be microaerophilic to facultatively anaerobic, the distal human gut is predominantly an anaerobic environment. However, microaerophilic niches can exist proximal to the gut epithelial surface, particularly within the mucus layer (53) (Table 1). The gut maintains a stable temperature of ∼37 °C, with a consistently moist environment, and the pH is buffered between ∼5.5 and 7. This pH balance is generally modulated by microbial fermentation products and bicarbonate secretions (54). Fecal residence times progressively increase along the colon (55), with longer colonic transit time resulting in a reduction in microbial biomass and diversity (56).

### Nutrients and crosstalk

Within the root rhizosphere, the microbiome is supplied by root exudates derived from photosynthates, sloughed root and border cells, and the mucilage surrounding plant roots (Table 1). Altogether, ∼25% of photosynthate is root products (57), which mediate plant health such as stress resistance, nutrient transport, reduction of soilborne disease, inhibition of nearby competitive plants, and nutrient mobilization (58–60). Similar to how the gut's mucous layer acts as a physical barrier, the root mucilage provides protection by forming a continuous hydrated matrix that prevents direct contact between the root and surrounding air pockets (61). Exudates are composed of both primary metabolites such as amino acids, sugars, and secondary metabolites such as flavonoids and coumarin (62–64), including naringenin (65), which recruits nitrogen fixers (diazotrophs) and together supplies their high C demands. Flavones enrich *Pseudomonas* to enhance N utilization and thus root growth (66) and induce nodulation by rhizobia (67), malic acid recruits plant growth–promoting rhizobacteria (PGPR) such as *Bacillus subtilis* and *Pseudomonas fluorescens* (68), tryptophan promotes colonization by indole-3-acetic acid supporting multiple PGPR (69), and scopoletin recruits *Pseudomonas* (70), among others. Microbial compounds produced in the rhizosphere can directly modulate plant immunity. For example, surfactin and fengycin production by *B. subtilis* and *N*-acyl-L-homoserine lactone production by *Serratia plymuthica* can trigger induced systemic resistance (ISR) in plants (71, 72).

Compared with the rhizosphere, the gut microbiome is less stable over short periods of time as nutrient inputs via ingestion vary temporally (Table 2). Here, carbohydrates (fiber) are metabolized by fermenting bacteria to produce short-chain fatty acids (SCFAs) (89). Among these, butyrate is particularly important for modulating inflammation and is an essential C source for colonocytes (90), while acetate and propionate positively influence homeostasis (91). Similarly, tryptophan is metabolized by gut bacteria into serotonin, which modulates gut motility and vagal signaling; indole derivatives that regulate gut barrier and immune responses; and kynurenine metabolites that influence neuroinflammation (92). Gut microbes such as *Lactobacillus* and *Bifidobacterium* produce gamma aminobutyric acid (GABA), a neurotransmitter that modulates depression and anxiety (93), while microbially synthesized catecholamines influence immune activation and epithelial permeability. A significant energy source for nonlumen (mucin-associated) microbes originates from the mucous secreted by the intestinal epithelial goblet cells. This mucous layer acts as a physical barrier that separates the lumen bacteria from the epithelial cells (94) and is rich in a variety of proteins such as mucin-2 (MUC2), which is accessorized with diverse glycans. These glycans serve to recruit commensal bacteria to the mucous layer (95, 96). However, pathogenic bacteria can also exploit this mechanism to decrease the proximity to the epithelial surface to initiate infection. Consequently, the type and availability of nutrients are spatially distributed both transversely down the colon and along the proximal–distal axis of the gut epithelial layer, shaping the recruitment of distinct microbial communities that contribute beneficial functions to the human host.

**Table 2:** Differences between the human gut and rhizosphere environments and microbiomes.

Feature	Human gut	Plant rhizosphere	Interpretation
Carbon source	Exogeneous, diet driven	Plant driven, autochthonous via photosynthate, soil carbon	Gut microbes rely on dietary intake while plants rely on photosynthate
Physical environment	Anaerobic, consistently moist, stable temperature, pH buffered	Variable O_2_ status and moisture, temperature fluctuations, more acidic	Rhizosphere influenced by external abiotic factors, gut is homeostatically maintained
Host immune systems and barriers	Innate and adaptive immune systems, IgA, IgG, IgM (73, 74)	Innate immune system (pattern recognition receptors), exudates (75)	More complex mammalian immunity, plants rely on PAMPs
Habitat/environmental influence	Relatively stable in adults, disruption through disease, diet changes, antibiotic use, etc. (76–78)	Highly temporally influenced, impacted by environmental stressors (natural or anthropogenic) (79–81)	Exposed linkages to a dynamic environment leads to lower stability in the root rhizosphere
Transmission mode	Vertical, mode of birth, early diet, environmental exposure (28, 82, 83)	Vertical through seed transmission then horizontal through soil (84, 85)	Both influenced by vertical and horizontal transmission though the rhizosphere can continually recruit from a much more diverse bank
Microbiome composition	Dominated by *Bacteroidetes*, *Firmicutes*, *Proteobacteria* (86, 87)	Dominated by *Proteobacteria*, *Actinobacteria*, *Firmicutes* (88)	Taxonomic profiles differ, especially at higher resolution due to host and environmental niches

In both ecosystems, bacteria produce density-dependent quorum-sensing molecules (e.g. *N*-acyl homoserine lactones) that enable bacteria to coordinate their behavior within a community setting, facilitating ecologically important functions such as biofilm formation, symbiosis, virulence, and extracellular enzyme production (97, 98). Similarly, tryptophan metabolism, which significantly influences microbial ecology and host physiology, shows striking functional convergence across these ecosystems (15). Despite differences in nutrient types and sources, both hosts coordinate analogous nutrient and signaling compound inputs to shape and sustain their associated microbiomes.

### “Core” taxonomic profiles in the gut and rhizosphere

For the rhizosphere microbiome, the proportion of shared microbes across individual plants of the same species or across species is expected to be smaller than the gut microbiome due to the niches soil provides that increase overall diversity (99, 100). Indeed, even when plant type is held constant (e.g. wheat), only ∼15% of all bacterial taxa (188 taxa) were shared across 20 wheat fields (101). However, the definition of a core microbiome varies widely, leading to little overlap across studies (102, 103). For example, a meta-analysis reported only 0.2–3.3% of amplicon sequencing variants (ASVs) qualified as core across major crops, with 8–15% universally shared (104). Thus, even after controlling for plant type differences, very large variations remain in the taxonomic composition of the rhizosphere microbiome. Comparisons are also influenced by the method of sequencing analysis, for example, using clustering operational taxonomic units (OTUs) versus ASVs.

For the human gut, interindividual variation among healthy individuals can be quite large, with only 30–40% of bacterial species shared and strain-level variation even greater (105). Geographical (ethnic) origin can significantly influence bacterial presence/absence, relative abundances, and diversity (106, 107). However, these effects can be overridden by dietary differences (108–111), even with small changes in diet over short periods (112). Host genetics also influences the compositional differences, although only a minority of gut bacterial taxa have been found to be “heritable” (36, 113). Additionally, a myriad of other factors influences gut microbiome composition to varying degrees (113), though a comprehensive discussion is beyond the scope of this manuscript.

### Core functional profiles in the gut and rhizosphere

Plants depend on microbial functions such as nutrient acquisition, growth promotion, and stress alleviation. As a consequence, some functional overlap across rhizospheres is expected, even when they are performed by different taxa. Accordingly, functional profiles across similar plant types are usually more conserved with high overlap in broad functional categories, especially pathways linked to core metabolism, signal transduction pathways, N metabolism, and biosynthesis of secondary metabolites (104, 114, 115). However, comparative data on core functional microbiomes across different plant species and soil types remain limited, as most rhizosphere studies focus on treatment effects within one, or just a few plant types and lack comprehensive shotgun metagenomic data. Thus, emerging evidence suggests that plant rhizospheres do harbor core microbiomes, though they are primarily defined by shared metabolic functions with high functional redundancy.

In humans, although there is large taxonomic variation between individuals, a larger shared component of functional genes and/or metabolic pathways is expected because individuals harbor similar metabolic niches (e.g. SCFA production and vitamin biosynthesis). Accordingly, functional profiles vary less than taxonomic profiles (116), however, as little as 38% of genes appear to be shared (86). Environmental factors, particularly diet, exert a strong influence on the shared functional profiles with 66% of annotated genes significantly different between the US and Malawian/Amerindian populations (36). Some specific pathways, such as Firmicutes-derived butyrogenesis (116), are more consistently conserved, likely due to the importance of this metabolic pathway in gut health. Collectively, these observations support the idea of functional redundancy within the human gut microbiome, a trait thought to promote resilience against perturbations (117) though the implications of functional redundancy for dysbiosis remain uncertain (118). Overall, there is a set of conserved bacteria at the phyla level, which aligns with the conservation of metabolic pathways that reflect core functions that support the clustering of microbiome members into enterotypes (119, 120) and reflect dietary and environmental influences rather than geography or ethnicity alone (121).

### Roles of microRNAs and snRNAs in the rhizosphere and gut

Plant microRNAs are small, noncoding RNAs that posttranscriptionally regulate gene expression via mRNA cleavage or translation inhibition (63). They play a significant role in hormone signaling, defense, and pathogen-induced transcription factors, where they can promote susceptibility or confer resistance to disease. These miRNAs function not only in the plant cells from which they originate but also can be transported into fungal and bacterial cells. Here, they can impart cross-kingdom silencing of RNA, which allows for rapid interactions between the plant and associated microbes (122, 123). The miRNA network involved in plant–pathogen interactions responds to both necrotrophic and biotrophic pathogens through salicylic acid (SA)- and jasmonic acid (JA)-dependent immune pathways as well as auxin signaling. For example, miR393 enhances SA-mediated defense and resistance to the bacterial pathogen *Pseudomonas syringae* in *Arabidopsis* (124). In potato, miR160/167 prevents pathogen-induced accumulation of auxin, which enhances resistance to *Fusarium oxysporum* (125). Many miRNAs target nucleotide-binding site leucine-rich repeat (*NBS-LRR*) genes, which are critical in both plant pathogen recognition and defense response (126), including miR482 in cotton against *Verticillium dahliae* (127) and miR472a in poplar against *Colletotrichum gloeosporioides* and *Cytospora chrysosperma* (128). MicroRNAs can also impact the composition of the rhizosphere microbial community. For example, miR393 increases the secretion of antimicrobial coumarins, which selectively enriches for *Flavobacterium*, *Chitinophaga*, and *Pseudomonas* (70, 124), and miR390 increases lateral roots and releases additional malate and amino acids, which shifts the community toward N-fixing *Bradyrhizobium* and *Azospirillum* (129). Similarly, prokaryotic sRNAs have been shown to regulate target genes, thereby influencing host metabolic pathways and defense responses in rhizosphere microbiome–plant interactions such as through *Trichoderma*-sRNAs enhancing plant immunity (130). Plant defense responses during arbuscular mycorrhiza fungal (AMF) formation through crosstalk between SA- and JA-dependent signaling pathways have been suggested to impact plant responses to later infection by plant pathogens (131).

Within the gut microbiome, members of the microbial community can influence host microRNA expression, while the host can also influence taxa, including pathogens, through microRNA production (132–134). These microRNAs are produced by gut epithelial cells and influence microbiome composition where they exert control over the transcription of genes involved in bacterial fitness (132). MicroRNA expression can be altered during the onset of various gut diseases such as Crohn's disease (135) and colitis (136). Many of the targets of these small RNAs were found to be involved in the regulation of immune responses and intestinal barrier maintenance (137). Excellent, in-depth reviews of the roles of microRNAs within the gut microbiome demonstrate their impact on various diseases such as cancer, obesity, etc. (133, 138, 139). The similarities across systems reinforce a developing theme; although the root and gut ecosystems differ in their biotic composition and abiotic influences, both have developed similar mechanisms to develop and maintain a beneficial microbiome. Here, a small taxonomic core harbors a larger shared repertoire of functions along with a molecular dialogue (e.g. microRNAs) that serves to support host health, resistance to disease, and resilience after infection.

## Dysbiosis: a shared pathological state

Dysbiosis, as a reference here to a disruption in the composition/function of a microbial community, can be characterized by a loss of beneficial taxa, reduced functional diversity, or the overgrowth of opportunistic organisms. Within the human gut, alterations in the composition of the microbiome have been linked to inflammatory diseases [e.g. inflammatory bowel disease (IBD)], metabolic syndromes, obesity, and susceptibility to pathogens (e.g. *Clostridium difficile*) (140–144). In the rhizosphere, there exist analogous disruptions that are most often triggered by environmental stresses, such as drought or heat, exposure to agrochemicals, or pathogen invasion, which can lead to reduced nutrient uptake, increased vulnerability to soilborne pathogens (take-all, *Fusarium* head blight), or weakened immune priming (145–149). Therefore, dysbiosis can compromise host fitness in both systems, not merely due to the presence of pathogens, but also due to the suppression or absence of protective microbial functions. In the following sections, we will summarize corollary causes of dysbiosis in both gut and rhizosphere systems and highlight their analogous causes.

### Rhizosphere microbiomes: disease impacts on dysbiosis and composition/function

Pathogen attack is a biotic stress that impacts the composition of the rhizosphere microbiome via either the plant immune response or the pathogen itself. However, determining direct causal relationships either to the altered immune “state” of the plant or via direct interactions with the pathogen is challenging (150). Examples of pathogens known to induce changes in rhizosphere composition include *F. oxysporum* (151), *Fusarium solani* (152), *Rhizoctonia solani* (153), and *V. dahliae* (154). Invasion typically results in a drop in rhizosphere alpha-diversity (155, 156). However, there are cases in which diversity may increase or not be altered at all (150). This reflects the complicated interplay of pathogen type, plant physiology and response, soil composition, infection stage, environmental factors, etc. that cannot be generalized. Pathogen infection may also impart a strong enough influence as to impact the assembly of the rhizosphere microbiome. This may be reflected in a decrease in sample intervariability in diseased versus healthy rhizosphere microbiomes due to strong pathogen-induced deterministic selection (157).

Further difficulties in assessing causal changes to the microbiome with pathogen invasion lie in the plant response. Known as the “cry for help” response, this alteration in plant exudates recruits beneficial taxa to reshape the community during disease (68, 119, 158). As such, over pathogen invasion/infection time, resident microbes may be displaced by those that are recruited. This makes separating a “diseased” from a “healthy” composition difficult as recruitment alters over disease progression, especially before the plants cry for help. Nevertheless, specific taxa that have been reported to respond to a cry for help or to be altered with disease progression include *Pseudomonas* spp. (159), *Stenotrophomonas*, *Microbacterium*, *Xanthomonas* (160), *Chitinophaga*, *Flavobacterium*, *Pedobacter*, *Variovorax*, *Rhizobium* (161), *Streptomyces* (162, 163), Comamonadaceae (164), and fungi such as *Penicillium*, *Trichoderma* and *Gliocladiopsis* (165), and *Chitinophaga* and *Enterobacter* (166).

Much of the current evidence used to categorize the presence of beneficial taxa in both systems is derived from *16S rRNA* gene or *ITS* gene amplicon surveys where the phylogenetic resolution allows for taxonomic assignments to be made at the genus level, at best. Given the substantial functional variability that can exist among strains within the same species (e.g. *Streptomyces* and *Trichoderma*), it can be extremely misleading to draw conclusions based on inferred function (167) at this resolution. As argued by Flores-Nunez and Stukenbrock (150), functional predictions should be approached with caution, particularly considering the limitations in reference databases that are largely based on human microbiomes (167). In contrast to the human microbiome, which has been more fully characterized using both culture-dependent and independent methods, robust functional linkages in the rhizosphere are less obvious and constrained by the greater diversity present in the soil habitat.

### Human microbiome: disease impacts on dysbiosis and composition/function

Analogous to the rhizosphere, the onset of disease in the gut is correlated with alterations in the composition/function of the microbial community, particularly through the depletion of “beneficial” microbes that regulate inflammation and synthesize health-promoting metabolites. For example, in inflammatory bowel disease, including both Crohn's disease and ulcerative colitis, reductions were observed in *Faecalibacterium prausnitzii*, which possesses anti-inflammatory properties and produces butyrate (168), *Roseburia hominis* and *Eubacterium rectale* (169), which are SCFA producers that maintain the integrity of the epithelial layer; and *Bifidobacterium* spp. (170) which impart anti-inflammatory effects. These reductions are often accompanied by increases in potentially pathogenic taxa such as *Escherichia coli*, associated with mucosal invasion in Crohn's (171), and *Enterococcus faecalis*, which is associated with mucosal damage (172). Other potential pathogens that increase in abundance, such as *Clostridium bolteae*, *Clostridium hathewayi*, *Bacteroides fragilis*, and *Enterobacter* species, have also been identified (173, 174). Irritable bowel syndrome presents another example of disease-associated microbial dysbiosis, which is characterized by decreases in *Bifidobacterium* spp. and *Lactobacillus* spp. (175), *Prevotella* and *Methanobrevibacter smithii* (176), as well as increases in Enterobacteriaceae, associated with changes in the motility of gut contents and some members of *Clostridium* cluster XIVa (177). Across a range of disease states, a consistent pattern of dysbiosis emerges, marked by the loss of SCFA-producing commensals and a concurrent rise in pathobionts and/or proinflammatory taxa. These shifts in the gut microbial community cause inflammatory impairment in the integrity of the epithelial barrier that can ultimately reinforce disease progression.

### Similar impacts of agrochemical and antibiotic usage

Pesticides play a role in agriculture analogous to the use of antibiotics in medicine; they are broad-spectrum biocides that kill both targets and some nontargets alike. Antibiotic use is associated with decreased microbial diversity and reduction in beneficial gut microbiota (178, 179) which allows for growth of pathobionts. Mirroring the human gut effect, certain agrochemicals result in the reduction of soil microbial richness, functional potentials, and PGPR which is often accompanied by a decrease in the natural disease-suppressive capacity of the soil (180). This loss of disease suppression is due in part to the disruption of antagonistic microbes that keep opportunistic pathogens at bay or the metabolic stress on the overall community. The archetypal human example is infection by *C. difficile*, which is normally kept in check by beneficial, competing anaerobes. Antibiotics disrupt this gut microbial barrier so that *C. difficile* can colonize epithelial niches and secrete enterotoxins. Similarly, outbreaks of Rhizoctonia root rot in cereals or *Pythium* damping-off in seedlings can occur in soils where agrochemical use has disrupted the natural microbiome (181, 182). Therefore, both the gut and the rhizosphere mechanistically lose their colonization resistance (183, 184). Both antibiotic and agrochemical disturbances can also drive the emergence of resistance traits. Antibiotic exposure increases the reservoir of transferable resistance genes in the gut microbiome (185), which can complicate infection management, while intensive pesticide exposure can select for resistant or pesticide-degrading microbes, which can further perpetuate the impact of agrochemical residues (186–188). Ultimately, in both cases, the outcome is increased susceptibility to disease, either directly from pathogen action (e.g. toxins) or indirectly through the loss of beneficial microbes.

## Dysbiosis and the immune system

Within both systems an attuned microbiome fosters immune equilibrium, whereas a perturbed microbiome warps the immune response toward either ineffective or harmful extremes (15). While both animals and plants rely on their associated microbiomes to deter disease, plants do so to a higher degree since they lack an adaptive immune system and thus immune memory. Plants defend themselves from pathogens through a multilayered innate immune system that is similar to that of animals. The primary layer is pattern-triggered immunity which is activated when plant surface receptors detect conserved microbe-associated molecular patterns (PAMPs if from pathogens) (189). These plant defenses are largely orchestrated by SA and JA, where SA is critical for resistance against biotrophic pathogens, which feed on living host tissue, and is a key component of systemic acquired resistance (SAR).

The gut microbiome, under normal circumstances, regulates excessive inflammation and maintains homeostasis (190, 191) through interactions between the gut microbiome and lumen. When the root microbiome is disrupted, particularly through the loss or reduction of beneficial microbial partners, pathogens face fewer barriers to infection. This microbial imbalance can open the door for opportunistic pathogens that would otherwise be suppressed. A recent work with tomato plants provides an example, where rhizosphere dysbiosis was induced by treating the soil with a broad-spectrum antibiotic, which reduced overall microbial diversity and abundance and created a dysbiotic state (not unlike antibiotic-associated inducement of *C. diff* in the human gut) (149). The dysbiotic tomato plants were significantly more susceptible to *Xanthomonas* leaf spot and developed more severe disease symptoms on their leaves, demonstrating the role of the root microbiome in enhancing systemic defenses, extending even to aboveground immunity.

Even though the underlying causes of dysbiosis differ between systems, the downstream consequences are similar. In both systems, there is a loss of beneficial microbes accompanied by an increase in pathogens that exploit disrupted niches. Interventions that aim to restore a healthy microbiome through amendments such as prebiotics and probiotics in the gut or biofertilizers and crop rotations that enrich beneficial rhizosphere communities represent a shared strategy to bolster host resilience.

## Implications for the treatment of dysbiosis

Recognizing the similarities in host–microbiome interactions across the plant rhizosphere and human gut has significant implications in the way that we can approach health and disease management (34). This insight emphasizes the adoption of microbiome-centered strategies that maintain or restore balance in the microbial community in composition and, more importantly, function. Central to the ability to restore a beneficial community is the broad use of probiotics and prebiotics in the gut. Similarly, in the rhizosphere, analogous approaches would be the use of microbial inoculants and soil amendments that increase microbiome diversity and abundances of beneficial microbial guilds.

### Gut probiotics and rhizosphere inoculants

Probiotics are live microorganisms that can confer a health benefit to the host. The first suggestions for the administration of live organisms for the treatment of diarrhea were by Henry Tissier in 1907 (192). Currently, most gut probiotics belong to a limited number of genera such as *Lactobacillus*, *Bifidobacterium*, *Lactococcus*, *Enterococcus*, *Propionibacterium*, *Saccharomyces*, and *Streptococcus* (193). These are proposed to or have been shown to ameliorate obesity (194, 195), provide antipathogenic activities (196), prevent necrotizing enterocolitis (197), regulate brain behavior (198), including depression and anxiety (199–201), and regulate mucosal inflammation (202–204). Significant effects have been found for a variety of diseases, such as pouchitis, infectious diarrhea, antibiotic-associated diarrhea, irritable bowel syndrome, ulcerative colitis, inflammatory bowel disease (205), celiac disease, and irritable bowel syndrome (206), among others. Antipathogenic activity by probiotics is thought to be due to the production of SCFAs, bacteriocins, and antimicrobial compounds, among others (207–211). Thus, there is convincing evidence that probiotic supplementation can improve disease by modulating the host microbiome in “unhealthy” individuals.

However, the efficacy of gut probiotics is dependent on their interactions not only with the host but also with the microbiome. For probiotic microbes to exert beneficial effects on the host, they must first overcome the competitive pressure exerted from the densely populated and well-adapted resident microbiota. Since the shared core microbiomes represent only a small percentage of the overall microbiome among individuals, the survival and persistence of probiotic strains must vary across people. Also, while there is significant data regarding the use of probiotics in diseased guts, randomized controlled trials show little evidence for a significant impact on the fecal microbiome composition in healthy adults (198). Probiotic strains often survive for a very short period only, with the transient colonization strongly shaped by the composition of host microbiome (212), and microbiome changes induced in healthy individuals may not persist (213).

An analogous approach to probiotic application in the rhizosphere involves the use of microbial inoculants, particularly PGPR (214, 215), which commonly include the genera *Bacillus*, *Pseudomonas*, *Azospirillum*, and *Rhizobium*, among others. The primary goal for their introduction is to improve plant health and productivity (216) through functions such as disease biocontrol (217), phosphate solubilization (218–220), phytohormone production, and N fixation (221–224). Specific biocontrol agents (BCAs) against a variety of soilborne pathogens have been adopted that utilize a variety of mechanisms to reduce plant infection (225, 226). Inhibition can be accomplished by direct antagonism, such as through the production of antibiotics, enzymes that degrade pathogen quorum-sensing molecules and effectors, such as biosurfactants, lipopeptides, or cell wall–degrading enzymes (227). Beneficial fungi such as arbuscular mycorrhizas, root endophytes, ectomycorrhizas, and yeasts can serve as BCAs. Antagonism may also occur through mycoparasitism, where a parasitic fungus penetrates fungal pathogen hyphae and extracts nutrients directly from the host (e.g. *Trichoderma*) (228, 229). BCAs may have the ability to confer systemic resistance to host plants through SAR or through ISR (230–232). Additionally, they can also induce modifications to the plant defense system, such as through upregulating defense-related genes in the JA pathway (233, 234). Plants can also be exposed to nonpathogenic strains of the same or different species within the pathogen genus, which can induce plant defense, termed “cross-protection” (235). Indirect antagonism, such as competitive exclusion, can also inhibit pathogen growth and/or migration to the root surface (236). Examples of successful applications of BCAs to plants include the fluorescent pseudomonads (*Pseudomonas* spp.) (237), *Azospirillum brasilense* (238), as well as *Serratia marcescens*, *Burkholderia phytofirmans*, and *Bacillus cereus*, among others (239, 240).

Just as probiotics may fail to establish if the gut environment is hostile or lacks necessary substrates, microbial soil inoculants may have difficulty in establishing themselves in soils that have low organic matter (OM), poor structure, microbial antagonists, or predation by protists (241, 242). Environmental factors may contribute to treatment failure such as pH, temperature, moisture, and nutrient availability. Thus, the efficacy of microbial inoculants is highly dependent on the utilization of a proper carrier to support sustained colonization of the root zone by allowing for the sufficient production of bacterial biomass needed to obtain the desired plant response. The addition of an inoculant alone often results in limited success. The inoculant must find an empty physical niche within the heterogenic soil, compete with the already-adapted resident bacteria, evade predation by soil fauna, and migrate to the root rhizosphere/rhizoplane. Therefore, addition with a carrier that provides a suitable microenvironment which includes physical protection and nutrients to sustain BCA growth over time is needed to increase success and to translate to a benefit at field scale (see Bashan et al. (243) for a review of inoculant formulations).

### Gut prebiotics and analogous soil carbon

Prebiotics are selectively fermented dietary components that are primarily nondigestible fibers and oligosaccharides that stimulate the growth or activity of beneficial microorganisms, in particular species of *Bifidobacterium* and *Lactobacillus*. The majority of evidence is based on treatments of irritable bowel syndrome (IBS), IBD, and ulcerative colitis. Many of these compounds can enhance microbiome diversity and stimulate the production of SCFAs that help maintain the integrity of the intestinal barrier (244, 245). Specific examples include resistant starch to increase butyrate production, modulate mucosal immunity, and reduce inflammatory markers (246), inulin which exhibits immunomodulatory effects (247), fructooligosaccharides (248), and xylo-oligosaccharides (249), which increase *Bifidobacteria* and partially hydrolyzed guar gum which enhances levels of SCFAs (250), among others. Many of these effects are directly on the gut microbiome by selecting for beneficial bacteria which may invoke immunomodulatory effects that can further alter the community without the need for direct probiotic supplementation.

Analogous to the use of fibers and oligosaccharides in the human gut, the rhizosphere microbiome can also be altered with the addition of a carbon source, such as OM. Soil health is directly impacted by a loss of soil OM (SOM) through a reduction in microbial biomass, taxonomic/functional diversity, the prevalence of PGPR (251), nutrient and water availability (252, 253), a fragmentation of microbial interactions (254), and increased susceptibility to plant disease with higher pathogen colonization success and diminished plant immune priming (255). Thus, the use of organic amendments, e.g. biochar, compost, manure and root growth promoters, serves as a functional analogs to prebiotics. These heterogeneous amendments improve soil structure, secondarily increasing water-holding capacity and nutrient cycling and provide diverse carbon substrates. Specific carbon compounds can recruit and enhance the populations of PGPR. For example, amino acids, humic substances, and organic acids enlarge populations of *Pseudomonas* and *Bacillus* (256 ), whereas composts enriched in cellulose and lignin promote fungal antagonists like *Trichoderma* spp. and other beneficial bacteria that produce antifungals (257). A meta-analysis found that composts and vermicomposts alone are ∼75% effective in controlling fungal disease, similar to humic substances (258). Some biochars promote disease suppression by supporting beneficial microbes, enhancing microbial competition and antibiotic production (259). The addition of specific C-compounds such as chitin can help recruit PGPR *Streptomyces* (260) as well as *Bacillus*, while decreasing plant pathogens (261) potentially by activating chitinolytic microbes (262). Thus, OM acts as oligosaccharides and fiber to the soil, recruiting beneficial microbes and imparting immunomodulatory effects on the plant immune system with specific carbon types, resulting in targeted effects within the microbiome.

### Synbiotic approaches to dysbiosis

A treatment that combines probiotics and prebiotics is termed a synbiotic (263), which are increasingly recognized for their potential in treating disease. Within the gut, synbiotics aim to synergistically enhance beneficial microbes as well as their metabolic activities by providing needed substrates. Examples include a mixture of six probiotic microbes combined with inulin and fructooligosaccharide for patients with dyslipidemia (264), a combination of *Bifidobacterium* and *Lactobacillus* combined with a *trans*-galactooligosaccharide for obesity (265), and mixtures of varying probiotics with either FOS or inulin for the treatment of metabolic syndrome (266). The mechanisms of action of synbiotics are identical to the combination of preprobiotics with the intent to improve the establishment success and prolong their longer-term residency. Again, many studies focus on diseased systems and not on the impact on an otherwise healthy microbiome, thus the true extent to which the beneficial bacteria remain within the gut remains largely unknown.

Similar to gut synbiotics, combining a C source such as amino sugars, organic acids, or polysaccharides with introduced taxa can create a more favorable environment for persistent colonization in the soil (267). Synthetic communities that use gnotobiotic systems (SynComs) that were reconstituted from cry for help associated microbes have reduced disease severity in *Arabidopsis* and tomato (160, 268). Other examples include the application of synthetic communities (SynComs) composed of bacteria and fungi for the suppression of *FOL* (*F. oxysporum* f. sp. *lycopersici*), the fungal pathogen responsible for fusarium wilt disease of tomato (269) and in the control of apple replant disease (270). Overall, in both rhizosphere and gut ecosystems, synbiotics offer the promise of a precision approach to engineering the microbiome by moving beyond single strain, inoculants to communities supported by respective nutrients that increase residence time.

### Holistic approaches to dysbiosis

Given that symbiotic mixtures are generally more effective than pre- and probiotics, fecal transplantation has gained prominence as a therapeutic intervention for a variety of disorders. Donor fecal material is screened for pathogens and infectious diseases and, most commonly, transplanted via a slurry directly into the colon terminal ileum, though the United States Federal Drug Administration (FDA) has cleared an oral FMT (fecal microbial transplant) capsule (Vowst). Clinical trials have shown cure rates >80% with some reporting >90% success after a single treatment for *C. difficile* infection (271–273). In some cases, the success rate is lower with oral administration with no significant difference versus placebo (274). FMT has also been used to significantly ameliorate depression and anxiety symptoms (275) with a meta-analysis (276) finding significant short-term improvement in depression, though long-term effects were less consistent (277, 278). Overall, fecal transplants are validated for the treatment of recurrent *C. difficile* infection and demonstrate enormous potential for managing neuropsychiatric and neurological disorders. This is all accomplished with the accumulated realization that transplanting beneficial microbes with their food source along with their environment and their commensal and competitive “neighbors” as a whole “neighborhood” to resolve gut-based disorders can ultimately confer the broadest and most sustainable impacts a dysbiotic system.

Can these same results be achieved by a soil transplant to resolve dysbiosis in the rhizosphere microbiome? In short, yes, this can be accomplished within a small-scale experimental system. However, it is not feasible in practice due to the enormity of soil that would have to be transferred to replicate diverse microbiomes associated with different microsites in soil. Despite this, it is possible to transfer disease suppressiveness (here to *Rhizoctonia* bare patch disease), enabled by the biotic component of the soil, into a disease-conducive soil by transferring 10% suppressive soil (w/w) (42, 279). However, soil transfer does not always impart suppression (280, 281). This should not be surprising since specific pathogen ecologies vary significantly, especially in relation to the wide array of abiotic and edaphic factors that soil microbes are exposed to as well as their connection to the broader bulk soil and its microbial inhabitants (282).

In summary, strategies that can be implemented to remediate dysbiosis in the rhizosphere and human gut exhibit conceptual parallels with practical differences. In both systems, increasing the abundances of BCAs or keystone commensals allows them to leverage their metabolic by-products and competitive advantages to both inhibit pathogen establishment and fortify host immune defenses. However, there remain issues that challenge the efficacy of these amendments. The gut environment confers colonization resistance that often limits the success of probiotic establishment through a combination of influences from the resident microbiome and host factors (immune, genetic, diet, age, etc.). In soil the inoculants face intense pressures from competition, predation, and abiotic stresses that can negatively influence their persistence, even with protective/supportive co-amendments. At the most holistic extreme, the transplantation of entire communities results in a high rate of treatment efficacy in the gut, while the analogous transfer of soil can also introduce beneficial impacts to both the microbiome and host health. However, this is impractical at field scales due to logistical constraints, which highlights a difference in feasible therapies between the rhizosphere and gut. Nevertheless, both systems converge around the paradigm that the repair of a dysbiotic microbiome requires an integrated approach of “seeding” the right microbes, “feeding” them to support their establishment and function, “modifying” the microsite environment that favors buildup of a beneficial microbiome and sometimes transplanting” whole microbial neighborhoods.

## Strategies for “disease management” and sustainability

Viewing the rhizosphere microbiome as a plant's “second genome,” similar to the human gut, encourages the concept of the host and its microbiome as an integrated evolutionary and functional entity in a holobiont perspective. In both host systems, the balance that is established between the host and microbiome illustrates the paradigm in which disease is not simply viewed as a two-player battle between host and pathogen. Rather, the holobiont is the true “entity” that fights off disease. This conceptional view urges a shift in both agricultural and medical practices toward evaluating the host system not only in the context of fitness but also within the broader context of ecosystem-level diagnostics and interventions. Using this conceptual parallel between systems offers a shared framework to interrogate for keystone species from both the community structure and functional perspectives as well as for functional redundancy and microbiome stability/resilience. Recognizing that dysbiosis is a common microbial syndrome that spans both environments not only can enrich our understanding of host-microbe ecology, including at the genetic level, but also can align strategies for restoring microbiomes across kingdoms.

These types of microbe-centered approaches also align with the core principles of resilience and sustainability. The case has been made across ecosystems that microbiomes are deteriorating due to a wide variety of practices at societal levels and that it is imperative that we take on the role of “microbiome stewardship” (283). In medicine, this supports the movement toward therapies that conserve microbiomes rather than the often-indiscriminate use of antibiotics (284–286). In agriculture, this means reducing our reliance on chemical fertilizers and pesticides in favor of interventions that are microbiome-informed and support disease-suppressive soil ecosystems (34, 215, 287–289). These interventions intend to restore microbial functional diversity and possibly, redundancy while re-establishing the host microbe equilibrium (241). Ultimately, health is rooted in the maintenance of a balanced, functional microbial ecosystem, supporting the One Health concept that describes strong connections between human, animal, and environmental health (290). This integrated perspective promotes cross-disciplinary innovation and underscores the fundamental role of microbial communities in the health and resilience of complex organisms, whether they be rooted in the soil or walking the Earth.
